# Deep learning enables automated localization of the metastatic lymph node for thyroid cancer on ^131^I post-ablation whole-body planar scans

**DOI:** 10.1038/s41598-020-64455-w

**Published:** 2020-05-08

**Authors:** MuthuSubash Kavitha, Chang-Hee Lee, KattakkaliSubhashdas Shibudas, Takio Kurita, Byeong-Cheol Ahn

**Affiliations:** 1 Graduate School of Advanced Science and Engineering, Hiroshima University, Hiroshima, Japan; 2 Department of Nuclear Medicine, School of Medicine, Kyungpook National University Hospital, Daegu, South Korea; 3 School of Electronics Engineering, Kyungpook National University, Daegu, South Korea

**Keywords:** Head and neck cancer, Whole body imaging

## Abstract

The accurate detection of radioactive iodine-avid lymph node (LN) metastasis on ^131^I post-ablation whole-body planar scans (RxWBSs) is important in tracking the progression of the metastatic lymph nodes (mLNs) of patients with papillary thyroid cancer (PTC). However, severe noise artifacts and the indiscernible location of the mLN from adjacent tissues with similar gray-scale values make clinical decisions extremely challenging. This study aims (i) to develop a multilayer fully connected deep network (MFDN) for the automatic recognition of mLNs from thyroid remnant tissue by utilizing the dataset of RxWBSs and (ii) to evaluate its diagnostic performance using post-ablation single-photon emission computed tomography. Image patches focused on the mLN and remnant tissues along with their variations of probability of pixel positions were fed as inputs to the network. With this efficient automatic approach, we achieved a high F1-score and outperformed the physician score (*P* < 0.001) in detecting mLNs. Competitive segmentation networks on RxWBS displayed moderate performance for the mLN but remained robust for the remnant tissue. Our results demonstrated that the generalization performance with the multiple layers by replicating signal transmission overcome the constraint of local minimum optimization, it can be suitable to localize the unstable location of mLN region on RxWBS and therefore MFDN can be useful in clinical decision-making to track mLN progression for PTC.

## Introduction

**Figure 1:**
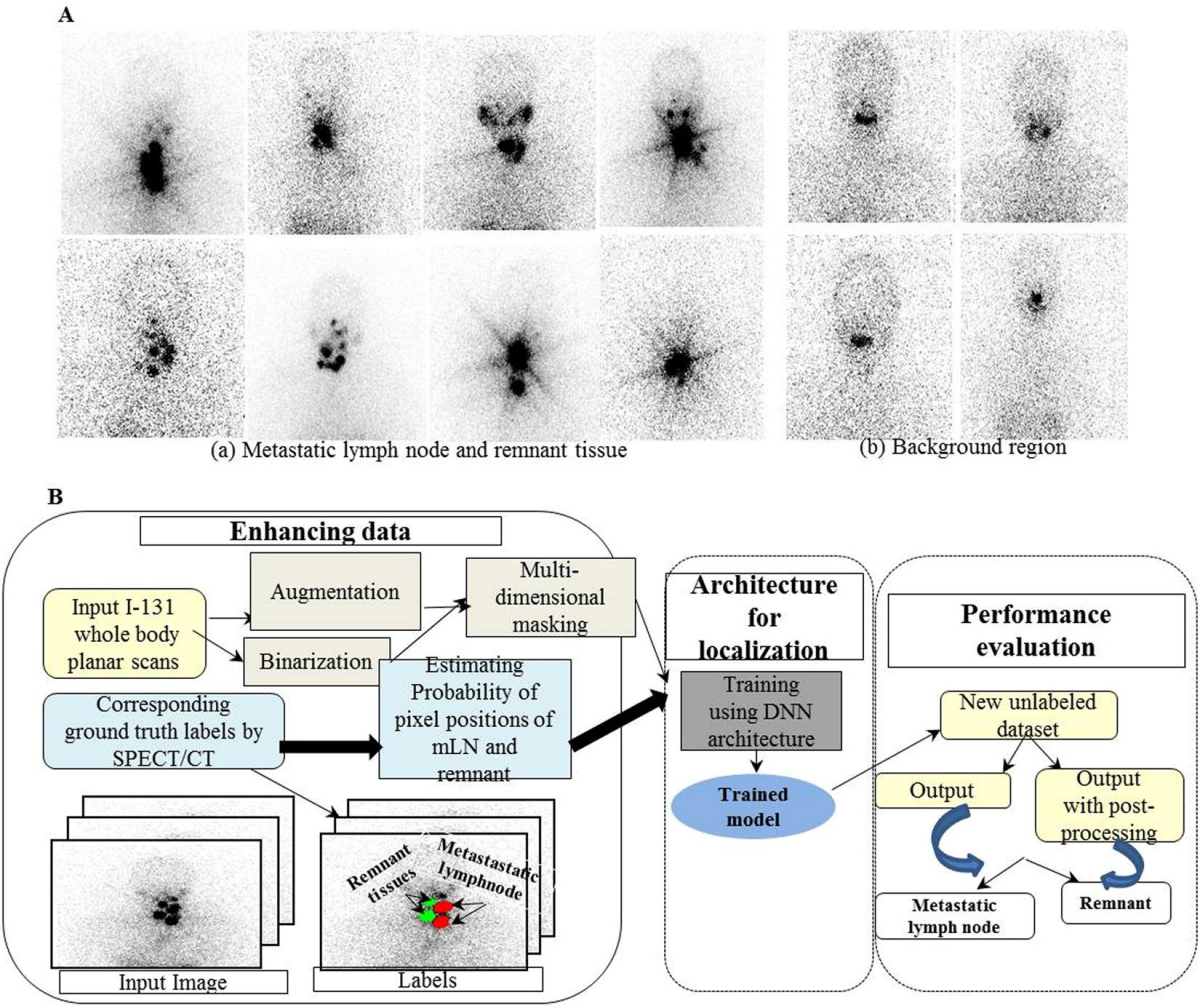
Original input ^131^I post-ablation whole-body planar scans affected by noise artifacts showing (a) both the metastatic lymph node and remnant tissue and (b) background oral cavity regions without the metastatic lymph node and remnant tissue (**A**). Proposed multilayer fully connected deep network for automatic recognition of the metastatic lymph node (**B**).

Lymph node (LN) metastasis is one of the major prognostic factors in patients with differentiated thyroid carcinoma (DTC)^1–3^. The prevalence of cervical LN metastases in DTC patients is in the range 20%–90%^4,5^. Post-ablation whole-body planar scans (RxWBSs) can visualize hidden, radioactive iodine (RAI)-avid metastatic LNs in thyroidectomized papillary thyroid cancer (PTC) patients and help in accurately staging the disease^6,7^. However, severe noise artifacts, scarcity of anatomical features on RxWBS, and the chance of RAI uptake make precise detection challenging, examples of which are shown in Fig. 1^8,9^. To overcome these problems, the assessment is usually based on three-dimensional single-photon emission computed tomography/computed tomography (3D SPECT/CT) data to detect the metastatic lymph node (mLN) on an RxWBS^9–11^. However, SPECT/CT-based differentiation and tracking the progression of mLN stages are tedious, expensive, and time consuming^12,13^. In addition, when the cancer treatment is successful, the mLNs decrease in size; thus, subjectively tracking the mLNs every time may result in errors. A practical solution to this issue is the automatic assessment of mLNs using RxWBS without any additional cost or radiation.

Several studies have focused on the higher recognition ability of RxWBS compared with using other imaging modalities for the detection of cervical mLNs in patients with PTC^14,15^. Ultrasound (US) has been a widely accepted first imaging tool for cervical mLNs^14^. However, small sized mLNs located in deep regions affected by thyroid tissues could influence low sensitivity. Furthermore, US is almost always used in a pre-operative condition and is believed to fail in distinguishing the hidden RAI-avid mLN found on post-ablation SPECT/CT^15^. The drawbacks of US have led to the use of other imaging techniques for PTC diagnosis. Several studies have encouraged the use of CT because of its higher sensitivity in locating the mLN compared with using magnetic resonance imaging^1,16,17^. However, CT is not recommended as a routine imaging tool for patients with PTC. Although the aforementioned imaging tools are considered superior in a number of instances, a ^131^I RxWBS is the only standard and possible modality for post-therapy imaging^18^. Nevertheless, it is complex, not always very precise, and sometimes provides a not clearly visible mLN position because of noise artifacts, thereby making accurate diagnosis challenging on RxWBS. Therefore, this study preliminarily attempts to adopt a learning-based pattern recognition methodology to automate the mLN for a precise PTC diagnosis.

Most of the automated studies in the literature have evaluated the use of various imaging modalities in predicting the status of LNs based on handcrafted features such as textures and morphological features in generating classification models^19,20^. Specifically, support vector machine-based methods have been frequently implemented and have subsequently produced better results for the discrimination of benign and malignant LNs^21,22^. Although conventional studies achieved highly satisfying results, they relied strictly on handcrafted features. In addition, identifying the most relevant features for the diagnosis required assistance. Furthermore, features such as boundary irregularity degree and shape may not be appropriate for mLN identification. To overcome all these problems, recent studies have focused on using a pre-trained neural network that automatically learns effective features to differentiate the nature of LNs^23,24^. However, the limitation of using deep learning is that a large number of datasets are required to increase the learning ability of the network. Several studies reported that deep convolutional neural networks (CNNs) required a minimum of 100 samples of each class for acceptable results^25,26^. In contrast, this study used a smaller number of mLN regions to develop the network. The multiple layers of the deep neural network scored competitive predictions in dealing with the complex and imbalanced class relationships of the samples by adding the number of hidden layers between the input and output layers^27,28^. We intended herein to propose a pixel-wise MFDN model that automatically extracts features from ^131^ I RxWBS and enables a computer-assisted automated technique for mLN localization. We are also interested in evaluating the performance of mLN localization with the competitive segmentation architectures because of its promising results in various medical imaging applications^29,30^.

The main challenges of this study are related to the highly complex RxWBS dataset along with the significantly limited number of training classes of the mLN region. To the best of our knowledge, no previous study has tackled the problem of the automatic distribution of mLNs based on RxWBS. This study aims to (1) develop and investigate whether the proposed MFDN is efficient for the automatic recognition ofunstable location of cervical mLNs on RxWBS, (2) evaluate the cross validation performance of the proposed system against the gold standard detection results using post-ablation SPECT/CT, (3) demonstrate the robustness of the proposed approach over the manual and CNN-based methods, and (4) confirm the feasibility of the automatic approach on a pilot study with an additional physician’s labels.

## Materials and methods

### Patients

This retrospective study was approved by the Institutional Review Board of Kyungpook National University Hospital, which waived the necessity of acquiring written informed consent. A total of 230 PTC patients who underwent total thyroidectomies, followed by RAI ablation at Kyungpook National University Hospital between March 2013 and December 2017, were reviewed.RAI ablation was performed at a median interval of 7 weeks (range: 4–14 weeks) after the total thyroidectomy.

### Data acquisition

The patients were orally administered with ^131^I (3.7 GBq, 100 mCi). A post-ablation ^131^I planar scan was then performed 4–5 days after ^131^I administration using a dual-head gamma camera equipped with 1.5875-cm NaI crystals and a 16-row multidetector spiral CT scanner (NM670; General Electric Medical Systems, Milwaukee, WI). SPECT/CT images were reconstructed with the Xeleris software (General Electric Medical Systems, Milwaukee, WI). No contrast medium was used during the procedure. All images from the RxWBSs and the post-ablation SPECT/CT were analyzed by nuclear medicine physicians. On RxWBS, the RAI uptake in the esophageal tract and salivary glands was considered to be physiological. The other focal tracer uptake on the background that was not compatible with the residual thyroid tissue or physiological activity was considered to be a pathological uptake. The SPECT/CT images were classified as (a) remnant tissue if the median foci were localized either in the upper thyroid bed in the thyroglossal duct remnants or in the lower thyroid bed or (b) RAI-avid LN metastasis if the focus was on a co-localized LN on CT.

### Data enhancement

**Figure 2:**
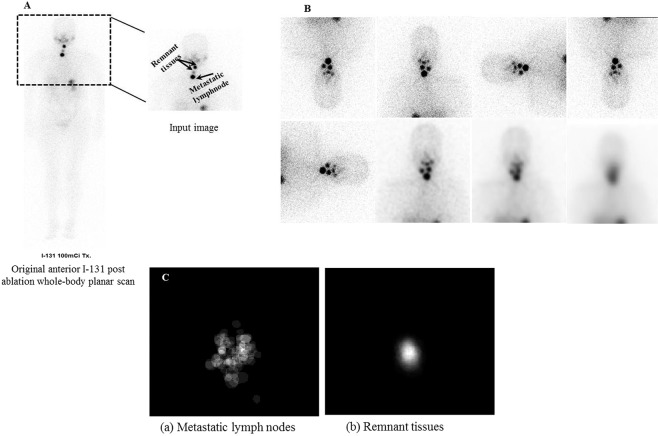
Cropped input image focused on the metastatic lymph node and remnant tissue from the original anterior ^131^I post-ablation whole-body planar scans (**A**). Enhancing the train data variations by augmentation using (top row from left to right) flip horizontal, flip vertical, rotate 90^0^, 180^0^, (bottom row from left to right) rotate 270^0^ and Gaussian kernels with varying sigma values (**B**). Estimation of the pixel positions in an image: (a) metastatic lymph nodes and (b) remnant tissues (**C**).

The RxWBSs were highly complex and usually affected by noise artifacts, and overlapped with the other physiological uptake regions (Fig. 1A). We proposed herein an automatic approach (Fig. 1B) to localize the mLN on RxWBS. In addition, directly training an original RxWBS with MFDN was not appropriate. We enhanced the training set by the augmentation method. Image patches of 230 × 230 px focused on mLNs, and remnant tissues were cropped from the original anterior image (Fig. 2A). For augmentation, each image is flipped along the horizontal and vertical axis, and rotated using an angle of 90^0^, 180^0^ and 270^0^. Augmentation with the gaussian filtering kernels with varying size σ, such as 2, 4, and 8 was computed which represented the different standard deviation of the gaussian distribution. The size of the kernel is based on the sigma value and hence square shaped kernel was used.The gaussian filtering estimated on each pixel, generated additional training sets with Gaussian pixel features^31^. Figure 2B shows examples of the data augmentation. The regions of interest (ROIs) on the gray-scale image were used by setting a threshold value of 160 px, which is a value determined by an experiment preserving the foreground pixels of the ROI. We calculated the label image to remove some unwanted objects such as physiological uptake and background. It generated a masked image with the desired object, where all pixels having the same value belonged to the same object. The resultant binarized image was then multiplied with the Gaussian kernel images, which produced multidimensional masked images with high-level representations of object pixels. The likelihood of a pixel position in an image being an mLN or remnant tissue (Fig. 2C) was calculated by normalizing the ground truth label values of pixels for all the training images using the following equations:

1 $$P({m}_{pl}|{R}_{rem})=\frac{1}{N}{\sum }_{i=1}^{n}G{T}_{i}$$

2 $$P({m}_{pl}{|R}_{met})=\frac{1}{N}{{\sum }_{i=1}^{n}GT}_{i}$$

where $P({m}_{pl}|{R}_{rem})$ denotes the probability of a pixel position being remnant and $P({m}_{pl}|{R}_{met})$ denotes the probability of a pixel position being an mLN, both at the pixel position ${m}_{pl}$. $G{T}_{i}$ denotes the ground truth labels of the training images *N*. The extracted high-level representation of the object pixels and the probability of the pixel positions of the mLN and remnant tissues were used as inputs for network training.

### Architecture design for localization

**Figure 3:**
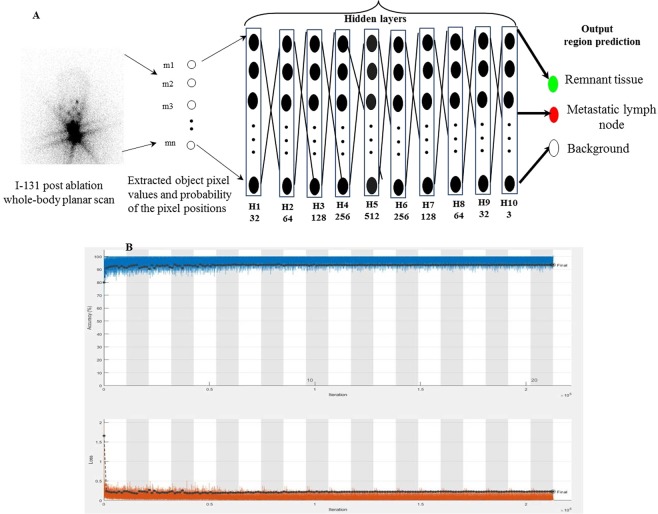
Multilayer fully connected deep network architecture for localization of the metastatic lymph node from thyroid remnant tissue (**A**). The learning curve for the best performing layers (10 fully connected hidden layers) on the training set (**B**).

Deep network architectures have been efficiently applied to image segmentation and classification to solve different tasks^32–34^. We proposed herein a MFDN model to recognize more complex and nonlinear relationships of pixels by incorporating the number of hidden layers between the input and the output. Our proposed architecture for localization was constructed with an image input layer, 10 fully connected hidden layers, and classification output layer (Fig. 3A). The number of nodes in the image input layer was equal to the number of pixel values associated with the image objects. The number of output layers was decided by the number of output classes. Similarly, in the intermediate hidden layers, each layer of nodes was trained on a discrete feature set based on the previous layers’ output. The number of neurons used in the fully connected hidden layer was increased by 32, 64, 128, 256, and 512 and then decreased by 256, 128, 64, 32, and 3. Combinations of the outputs from each hidden layer were executed by a linear function followed by a nonlinear transformation of the weighted sums. Three different nonlinear activation functions, namely, logistic sigmoid $s(r)=1/(1+\exp (\,-\,r))$, hyperbolic tangent $\tanh (r)$, and rectified linear unit (ReLU) $s(r)=\,\max (0,a),$ were applied to train our network model. The output of node *p* in layer $k+1$ indicated by ${Q}_{p}^{(k+1)}$ was constructed from the image inputs ${m}_{1}^{(k)},\,{m}_{2}^{(k)},\mathrm{..}.,{m}_{n}^{(k)}$ as follows:

3 $${Q}_{p}^{(k+1)}=s({r}_{p}^{(k+1)})=s({\sum }_{t=0}^{{n}_{1}}{w}_{pt}^{(k)}{m}_{t}^{(k)})$$

where *k* = 0, …, *K* and *p* = 1, …, *n*_*k*+1_. The linear combinations of the outputs are represented as ${r}_{p}^{(k+1)}$, and ${w}_{pt}^{(k)}$ denotes the weights of the linear function. The total number of hidden layers and the total number of nodes in the layer are represented by *K* and *k*, respectively. The bias node in layer *k* is denoted by ${m}_{0}^{(k)}$with a value of 1. ReLU was observed to be optimal because it was faster and produced robust performance among the other activation functions. After the fully connected layers were completed, the prediction map related to the likelihood of the pixel matching to a specific ROI class was obtained from the output vector of the class scores using the pixel-wise softmax cost function defined as

4 $${P}_{i}(v)=\left[\frac{{z}^{vi}}{{\sum }_{r}^{T}{z}^{vr}}\right]$$

where ${P}_{i}(v)$ is the probability that the pixel assigns the output vector *v* that corresponds to the ROI *i*, and *T* is the total number of ROIs. Classes (such as mLN, remnant tissue and background) were labeled on the ground truth datasets as 1, 2 and 0, respectively.

**Figure 4:**
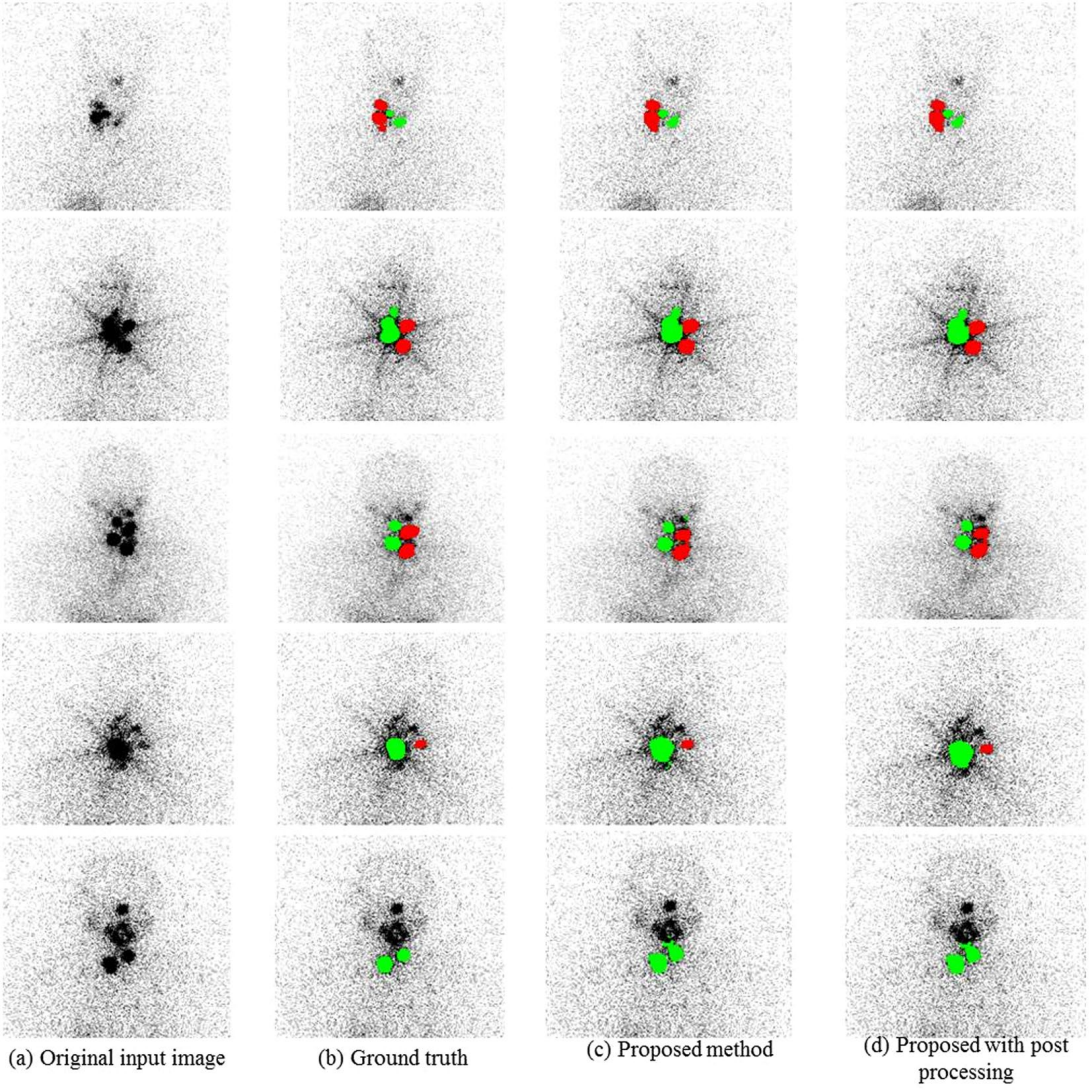
Localization results of the metastatic lymph node (red) and remnant tissue (green). (**a**) Original input ^131^I post-ablation whole-body planar scans, (**b**) true segmentation results by SPECT/CT, (**c**) proposed method, and (**d**) proposed method with post-processing.

The dataset was randomly divided into 60%, 20% and 20% for train, validation and test, respectively. The network used the augmented classes along with the original classes by increasing the training data variations. It trained on the train data while evaluating the fitness of the model on the validation data with fine-tuned optimal parameters to minimize the overfitting of the model. The number of epochs set to train the model was 20 that possess 212760 maximum numbers of iterations throughout the training while keeping 10638 iterations per epoch.The generalization of the model complexity was evaluated by setting to shuffle the train data before each training epoch and shuffle the validation data before each validation network frequency of 1200 iterations. To find the best model the network was trained by increasing the number of fully connected hidden layers from three to 10. We used Adam optimizer for cross-entropy minimization with a batch size of 64 and the learning rate of 0.001. The initial learning rate is updated every 10 epochs by multiplying with drop factor 0.1, which decreased the learning rate according to the piecewise linear method. We set weight decay using L2 Regularization of 1.0000e-04 and gradient moving average decay rate of 0.9000 and denominator offset epsillon 1.0000e-08 in the network parameter updates to avoid division by zero. The fitness of the model was learned from the validation data by minimizing the training error and maximizing the accuracy between the detected and true segmentation labels. On the basis of the learning curve, the best model with the number of fully connected hidden layers of 10 was identified and showed a sufficiently higher ability in segmenting the mLNs from remnant tissues with high accuracy (93.49%) and low error value (0.237), implies the model efficiently fits the datasets (Fig. 3B). We followed the post-processing method for the removal of incorrect regions, especially the overlapped regions of remnant tissue with the oral cavity regions. The size filtering of 30 pixels was chosen by an experiment and applied on the segmented output image. It removed the small incorrect regions. Figure 4 shows the localized final output regions from the proposed approach. The network was implemented using Matlab R2019b with 16 GB memory of NVIDIA GTX TITAN. The proposed segmentation network took 6.5 hours to train because of its large number of network parameters and test one RxWBS image in 1.3 s.

### Experimental setup

#### Comparison methods and evaluation metrics

The proposed automatic method was evaluated by comparing its output to the ground truth segmentation using post-ablation SPECT/CT. Network training and testing included mLNs, remnant tissues, and background regions without either mLN or remnant tissue. The localization performance of the proposed, proposed with post-processing, and physician’s manual methods in detecting the mLN, remnant tissue, background, and overall regions was evaluated. Manual detection without ground truth was performed by the same physician, who prepared the ground truth labels for the mLN and remnant detection. Network performance and manual detection were validated using precision, recall, and the F1-score. We also computed the Dice similarity coefficient (DSC), which is the most widely used parameter for measurement of the agreement of two segmentations (i.e., G and H). It is defined as$2|G\cap H|/(|G|+|H|)$, with a range of [0, 1]. The higher the DSC value, the better the overlap between the two segmentations. The generalization performance of the proposed architecture in recognizing mLN, remnant tissue, background and overall was evaluated using a five-fold cross-validation by partitioning the datasets into *k* folds (*k* = 5). The network was trained on *k*–1 fold with one held back, and tested on the held back fold. It continued five times separately by using different members of the training and testing data that includes compositions different from those of the other experiment.

We conducted a pilot study using 30 independent RxWBSs with highly unbalanced classes of mLNs (9) and remnant tissues (60). None of the 30 RxWBSs in the pilot study were included for training and testing the network. For the pilot study, we compared the performance of the proposed approach with the manual segmentation labels of two additional physicians in localizing the mLN from the remnant tissues. The physicians were blind to the patients’ clinical history and pathology and independently evaluated the RAI-avid LN metastasis and remnant tissue on the RxWBSs. The statistical significance of the observed differences of the performance metrics between the proposed method and the physicians was evaluated using a two-tailed, paired t-test, for which a *P* value of 0.05 was considered significant (Minitab 19.0.2 Statistical Software). Furthermore, we were interested in comparing the performances of mLN localization from the thyroid remnant tissue regions of our proposed model over the CNN-based architectures such as, 2DU-net^30^ and SegNet^35^. Given the training dataset, the CNN architectures were learned by minimizing the training error (cross-entropy error) between the detected and true segmentation labels using the same optimized parameters of the proposed network.

## Results

**Figure 5:**
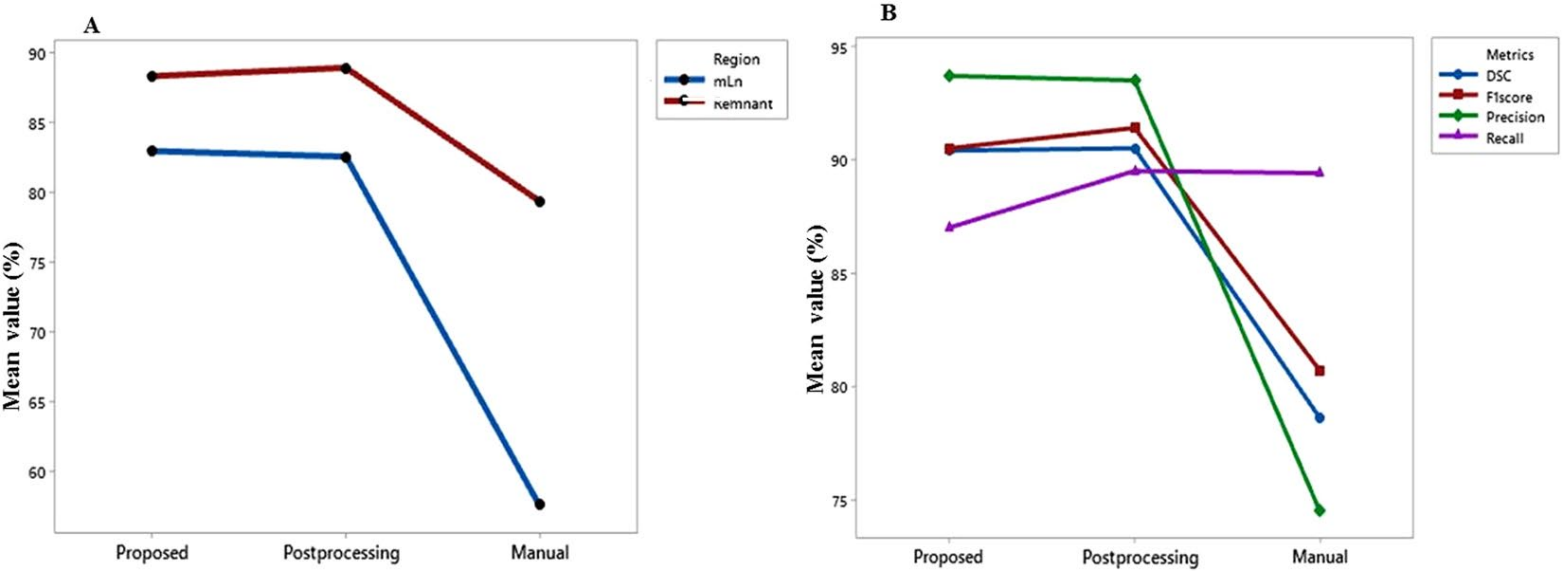
Performance comparison among the proposed, proposed with post-processing, and manual methods for localization of the metastatic lymph node and remnant tissue in terms of mean dice similarity coefficient (**A**). Overall performance comparison among the proposed, proposed with post-processing, and manual methods for localization of the metastatic lymph node, remnant tissue and background in terms of precision, recall, F1-score and dice similarity coefficient (**B**).

RxWBSs were efficiently constructed for the train (1794), validation (46) and test sets (46) with the proposed automatic approach. The augmentation technique was carried out only on the training set. Thus the training set consisted of 2366 remnant tissue regions and 478 mLN regions. The validation and test set included 56 and 59 remnant tissue, respectively and 12 and 10 mLN regions, respectively. Table 1 demonstrates the mean values of the performance measures of the proposed method without or with post-processing and the physician’s manual method in evaluating the mLN, remnant tissue, background, and overall regions on the test scans. Compared with the manual detection method in localizing the mLN, the automatic methods (i.e., proposed and proposed with post-processing methods) achieved the highest ranges of mean precision (84.7%–85.3%), mean recall (77.8%–84.2%), and mean F1-score (82.3%–84.4%). Similarly, these methods showed the highest recognition rate in localizing the thyroid remnant tissue compared with the manual method. Manual detection without ground truth showed low to moderate performance in detecting both the mLN and the remnant tissue, demonstrating that the proposed MFDN can learn to be more descriptive and better classify between regions compared with the manual method. Figure 5A demonstrates the differences of the DSC using the three methods evaluated herein in differentiating the two groups of mLN and remnant tissue. The diagnostic performances of the proposed method with or without post-processing were almost similar. It is also confirmed in the overall differences of the precision, recall, F1-score, and DSC using the three methods evaluated herein in differentiating mLN, remnant tissue and background regions (Fig. 5B). The post-processing method efficiently removed some incorrect regions that overlapped with the remnant tissue; however, it did not improve diagnostic performance. Thus, the MFDN localization algorithm without post-processing is sufficient to differentiate the mLN from the thyroid remnant tissues. The generalization complexity of the proposed MFDN is also confirmed with the results of the five-fold cross validation performance in evaluating the mLN, remnant tissue, background, and overall regions (Table 2). The five-fold cross validation performance in evaluating the mLNs from remnant tissues revealed model’s generalization error values (0.2731, 0.2018, 0.3010, 0.2145, 0.2932, respectively) denoting the optimal model complexity with low bias and variations in locating mLN for PTC.

**Table 1:** Comparison of the performance of the proposed method without and with post-processing and the physician’s manual method using post-ablation SPECT/CT.

Region	Method	Pre (%)	Rec (%)	F1-score (%)	DSC (%)
mLN	Proposed	85.3	77.8	82.3	83.0
	Proposed with post-processing	84.7	84.2	84.4	82.6
	Manual	51.2	76.5	61.3	57.5
Remnant tissue	Proposed	95.9	84.3	89.7	88.4
	Proposed with post-processing	96.0	84.5	89.9	89.0
	Manual	73.0	92.4	81.6	79.4
Background	Proposed	99.93	99.1	99.5	99.9
	Proposed with post-processing	99.97	99.9	99.9	99.9
	Manual	99.30	99.26	99.28	99.0
Overall	Proposed	93.7	87.0	90.5	90.4
	Proposed with post-processing	93.5	89.5	91.4	90.5
	Manual	74.5	89.39	80.73	78.6

mLN: metastatic lymph node; Pre: precision; Rec: recall; DSC: Dice similarity coefficient.

**Table 2:** Five-fold cross validation performance of the proposed method using post-ablation SPECT/CT.

Region	Five-fold	Pre (%)	Rec (%)	F1-score (%)	DSC (%)
mLN	Fold 1	87.0	88.0	87.5	86.7
	Fold 2	82.9	83.2	83.0	80.2
	Fold 3	90.1	69.7	78.6	79.3
	Fold 4	90.0	70.2	78.4	77.0
	Fold 5	72.0	70.4	71.1	71.5
Remnant tissue	Fold 1	97.0	77.5	86.2	87.1
	Fold 2	88.2	89.0	88.5	90.0
	Fold 3	98.3	81.0	88.8	88.0
	Fold 4	97.0	72.8	83.2	85.7
	Fold 5	98.0	79.0	87.5	88.0
Background	Fold 1	96.0	94.3	95.1	97.0
	Fold 2	99.5	100.0	99.7	99.3
	Fold 3	100.0	99.4	99.7	99.7
	Fold 4	99.0	99.2	99.1	99.0
	Fold 5	100.0	100.0	100.0	99.9
Overall	Fold 1	93.3	85.1	88.9	88.1
	Fold 2	90.9	90.7	90.8	90.5
	Fold 3	96.1	83.4	89.0	89.0
	Fold 4	92.3	82.1	86.6	87.2
	Fold 5	92.3	83.1	87.3	87.7

mLN: metastatic lymph node; Pre: precision; Rec: recall; DSC: Dice similarity coefficient.

**Figure 6:**
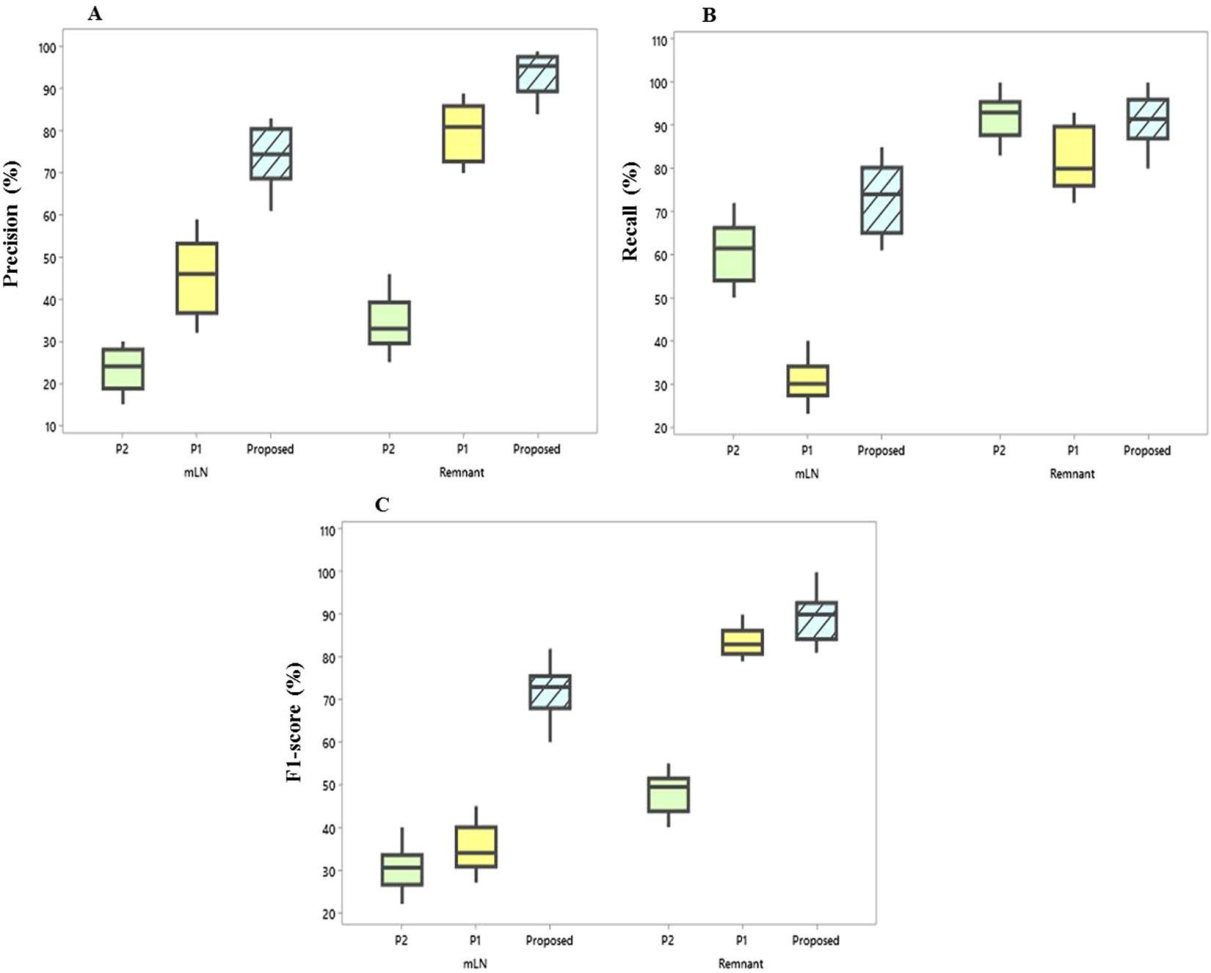
Box plot distributions and comparison of the proposed and two physicians represented as P1 and P2 for localization of the metastatic lymph node and remnant tissue on the pilot study dataset in terms of precision (**A**), recall (**B**), and F1-score values (**C**).

**Figure 7:**
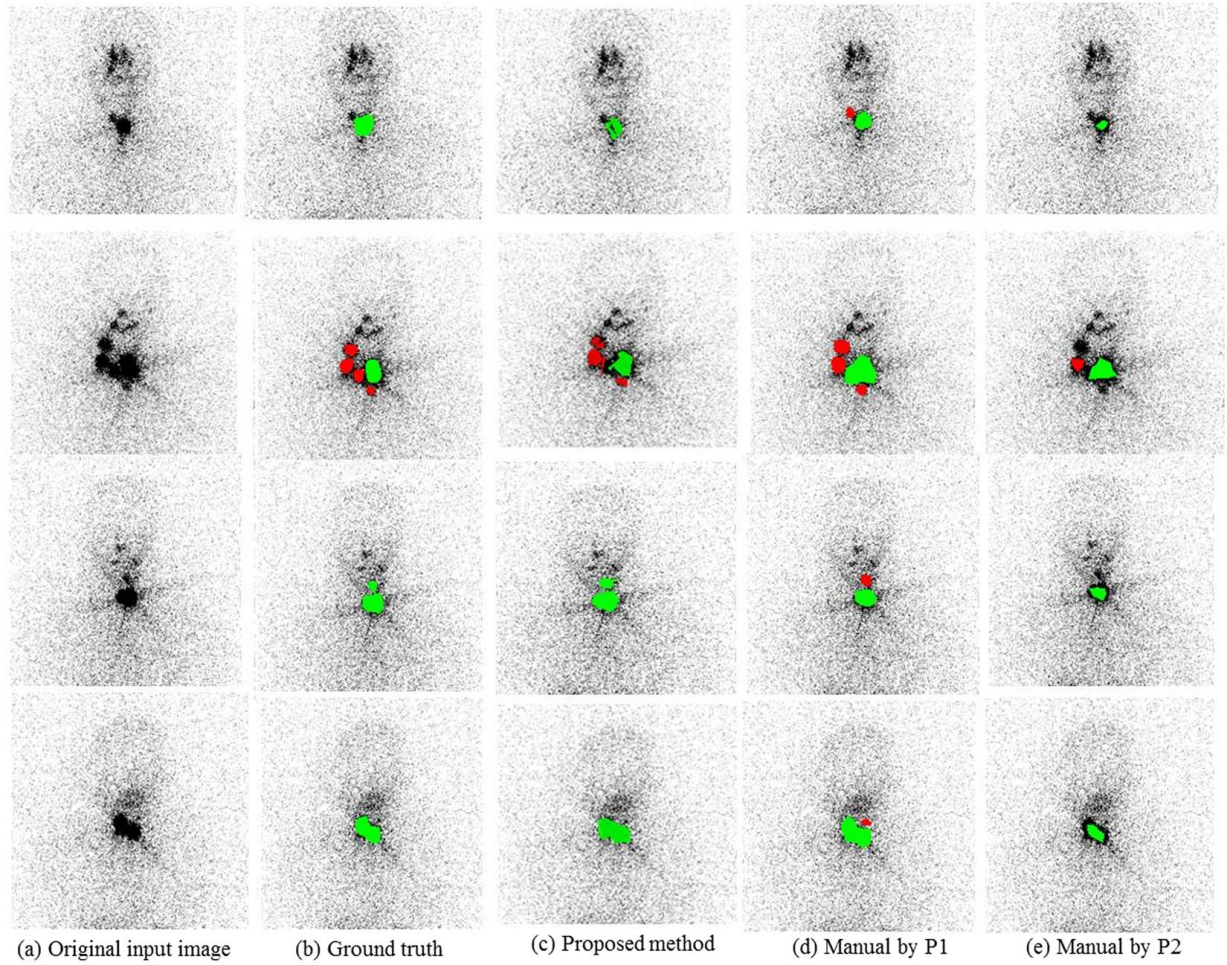
Localization results of the metastatic lymph node (red) and remnant tissue (green) using the pilot study dataset. (**a**) Original input ^131^I post-ablation whole-body planar scans, (**b**) true segmentation results by SPECT/CT, (**c**) proposed, (**d,e**) two physician’s manual labels represented as P1, and (**e**) P2.

Table 3 confirms the feasibility of the proposed approach from a clinical standpoint on an independent dataset and demonstrated significantly high performance measures in diagnosing mLNs compared with the manual segmentation labels of an additional two physicians represented by P1 and P2 in the table. The box plot presents the distribution of the precision, recall, and F1-score metrics of the pilot study dataset in detecting both mLN and remnant tissues (Fig. 6A–C). For utilization in clinical settings, the recall of an analysis should also support clinical decisions. From this point of view, the recall values in detecting the mLN by physicians, which varied between 28.9% and 59.2% via manual discrimination, may not be adequate. The differences between the mean precision and the mean F1-score values of the proposed and physician’s methods in detecting the mLNs were statistically significant (*P* < 0.001). The observed mean values in recognizing the remnant tissues with a precision of 97.5%, a recall of 90.0%, and an F1-score of 93.6% using the proposed method were high. The difference between the mean F1-score value of MFDN and P1 was not statistically significant (*P* = 0.29); however, the difference was statistically significant (*P* < 0.001) with P2. The imperfect agreement between the physician manual labels indicated the superimposition of the RAI uptake areas of the remnant tissue and the high noise that makes the manual interpretation challenging (Fig. 7). The consistencies between the SPECT/CT-based ground truth on each RxWBS and the proposed and manual segmentation contours of the two physicians were summarized using DSC in Table 3. The DSC value of the proposed method for mLNs over their ground truth of SPECT/CT was 73. 0%, this is almost 44.0% higher than the manual contours of the physicians. No significant difference was found between the manual contours of the physicians (*P* = 0.253) in recognizing mLNs on RxWBSs.

**Table 3:** Pilot study datasets showing the performance metrics and significance results of the proposed approach and each physician expert to localize the metastatic lymph node and remnant tissue.

Method	Pre (%)	Rec (%)	F1-score (%)	DSC (%)
**mLN**				
Proposed	74.2^**a^	73.0^*a^	73.5^**a^	73.0^**a^
P1	42.59^**b^	28.92^**b^	34.45^**b^	29.92^**b^
P2	20.89^*c^	59.21^*c^	30.88^# # c^	25.96^# # c^
**Remnant tissue**				
Proposed	97.5^**a^	90.0^# # a^	93.6^**a^	94.7^**a^
P1	82.80^# # b^	80.23^# # b^	81.49^# # b^	79.71^# # b^
P2	34.51^# # c^	95.50^# # c^	50.70^*c^	47.22^**c^

mLN: metastatic lymph node; Pre: precision; Rec: recall; DSC: Dice similarity coefficient; P1: physician 1; P2: physician 2.

*P < 0.05, ^##^P > 0.05.

**P < 0.001.

^**a**^Comparison between proposed and P2.

^**b**^Comparison between proposed and P1.

^**c**^Comparison between P1 and P2.

The diagnostic performance in differentiating the mLN and the thyroid remnant tissue using U-net architecture showed precision values of 66.4% and 96.5%, recall values of 48.6% and 84.8%, F1-scores of 56.0% and 90.3%, and DSC scores of 57.7% and 89.6%, respectively. The diagnostic performance in differentiating the mLN and the thyroid remnant tissue using SegNet architecture revealed better performance by utilizing more global contextual features than U-net and shows high precision values of 77.3% and 88.1%, recall values of 62.1% and 94.9%, F1-scores of 68.9% and 91.4%, and DSC scores of 70.0% and 91.0%, respectively. For the mLN evaluation, both the U-net and SegNet architectures showed a low performance with high error values of 0.605 and 0.379, respectively. It showed inability and optimization instability in locating the mLNs. However, it may be possible to increase the capacity of those networks on RxWBS datasets by setting the network weight initialization towards the mLN region at the start of the training model using optimized loss and sampling methods. Although the proposed sequence of fully connected layers consists of a large number of parameters, the multiple layers by replicating signal transmission in MFDN overcome the constraint of local minimum optimization and thus it can be suitable to localize the unstable location of mLN region on RxWBS datasets.

## Discussion

The accurate detection of mLNs on RxWBS is essential in setting up management and follow-up plans for PTC patients^3^. However, the highly complex RxWBS with severe artifacts and overlapped ROIs along with the limited amount of mLN data for training were key challenges in this study. Our proposed MFDN on RxWBS involved no manual interaction. Furthermore, the simplified description of the learning-based approach facilitates efficient automated localization of mLNs in clinical practice. To the best of our knowledge, this is the first study to develop successful adoption of the deep neural network architecture on RxWBSs, which is capable of recognizing the mLN and the remnant tissue. The promising experimental results of our proposed MFDN without post-processing displayed robust performance measures over the manual detection method. With this efficient automatic approach, we achieved a high F1-score (82.3%), which was 21.0% higher than that of the manual contour detection method. The performance of our approach in locating mLN regions was always higher than those achieved via the physicians’ manual segmentation labels; however, the performance remained robust in locating the thyroid remnant tissue. Similarly, the CNN showed moderate performance in mLN detection and robust performance in thyroid remnant tissue detection. Our results demonstrated that the MFDN model is a useful tool for RxWBS because it especially improved the generalization performance in localizing the mLN with the increase in the number of hidden layers.

The approaches extracted hand-engineered features in the image, and the designed complicated quantification methods for LN localization gave recall rates of 82.0%^16^, 60.0%^17^, 86.0%^36^, and 90.0%^37^. However, they were not appropriate for the detection of mLNs, which is often indiscernible from adjacent tissues with similar gray-scale values. In addition, compared with some existing deep learning-based techniques for the automated identification of PTC, our method demonstrated the use of a simple multilayer stacked framework. Li *et al*. developed a detector network based on the CNN, particularly for PTC on US, and reported a recall rate of 93.5%^38^. Their detector cannot identify cancer regions if a particular layer fails to extract whole features in the image. Halicek *et al*. developed a CNN-based model for classifying head and neck cancer on a small subset consisting of 21 hyperspectral images and reported a recall rate of 86.0%^39^. Ma *et al*. concatenated several network training stages on the basis of the CNN for the thyroid and achieved a recall rate of <90.0% from the SPECT images^40^. However, the aforementioned approaches implemented different learning designs and imaging modalities for thyroid cancer, which were not appropriate in directly gauging our outcome. Though the combination of radiomic features and CNN served a high performing tool for classifcation task, directly accessing the raw data may not be suitable for the segmentation of regions on homogeneous classes with noisy background^28,41^. The CNN-based architecture evaluated herein demonstrated lower performance in differentiating the mLN than our approach. It may be due to the patchwise splitting of minor variations of image objects using CNN architecture prone to loss certain degrees of required information^28^. Hence, it may poorly generalize the mLN location on the unseen data. Hence an experiment using an explicit regularizer with an appropriate loss function could be helpful to improve the CNN networks on RxWBS datasets. This is beyond the scope of the current study. As mentioned earlier, the purpose of our study was not to deeply explore the learning strategies of the imbalance problem, but to make a preliminary attempt to adopt a deep learning-based technique for the accurate localization of the mLN for PTC. Our approach offered the advantages of a single deep framework with highly acceptable performance in segmenting mLN from noisy RxWBSs.

Additional evaluations were performed during this study, especially focusing on clinical perspectives. The validation and comparison of the discrimination results between the automatic deep learning approach and the manual contour labels were intriguing. As can be seen in Fig. 6, the performance of manual discrimination indicated more variation. It also showed lower performance than the proposed automatic technique. The DSC of the proposed automatic method was higher than those of the physicians; hence, with a lack of ground truth, the physicians were challenged in accurately identifying the mLN on RxWBSs^15,18^. Furthermore, the superimposition of the RAI uptake areas of remnant tissue and the high noise affecting manual interpretation were observed with the number of false positives and false negatives (Fig. 7). The automatic approach utilized the intensity statistics of entire image patches containing the ROIs and surrounding tissues, thereby reducing errors, which human observers hardly understand. The results were encouraging, but there is still much improvement required in the system in terms of stability by including a large number of mLN patterns in the training. Therefore, we expect that our proposed deep learning segmentation tool could be used as a support in clinical practice and increase a physician’s performance by reducing the interobserver variation, interaction time, and workload required for mLN recognition.

The limitation of this study is that a small amount of representative training data was used to build the learning model. The mLN patterns on the datasets seemed to not sufficiently comprise the training set. Additionally, the RxWBSs were collected using the same scanner and with the same acquisition protocols for effective training. Hence, our proposed approach must be optimized using a large number of mLN input patterns with different RxWBS scanners and acquisition protocols. Finally, different CNN-based neural network architectures with various learning strategies will be evaluated in future.

## Conclusions

In the approach proposed herein with the high noise levels of the ROIs, RxWBSs were efficiently constructed using the MFDN model, thereby offering excellent diagnostic performance to recognize the mLNs. Our model is simple, cost-effective, and does not require expert knowledge for successful implementation. Compared with the manual contour labels from the physicians and the CNN-based segmentation network, our proposed MFDN model gave highly acceptable localization performance, thereby acting as a promising substitute for the precise discrimination of mLNs and remnant tissues on RxWBSs. The experimental results strongly suggest that the proposed automated deep learning model can be used as a support in clinical practice to track the progression of mLNs for effective cancer treatment.
