# Author Correction: Deep learning enables automated localization of the metastatic lymph node for thyroid cancer on ^131^I post-ablation whole-body planar scans

**DOI:** 10.1038/s41598-020-68538-6

**Published:** 2020-07-09

**Authors:** MuthuSubash Kavitha, Chang-Hee Lee, KattakkaliSubhashdas Shibudas, Takio Kurita, Byeong-Cheol Ahn

**Affiliations:** 1 Graduate School of Advanced Science and Engineering, Hiroshima University, Hiroshima, Japan; 2 Department of Nuclear Medicine, School of Medicine, Kyungpook National University, Kyungpook National University Hospital, Daegu, South Korea; 3 School of Electronics Engineering, Kyungpook National University, Daegu, South Korea

Correction to: *Scientific Reports* 10.1038/s41598-020-64455-w, published online 08 May 2020

The original version of this Article contained an error in Affiliation 2, which was incorrectly given as ‘Department of Nuclear Medicine, School of Medicine, Kyungpook National University Hospital, Daegu, South Korea’. The correct affiliation is listed below:

Department of Nuclear Medicine, School of Medicine, Kyungpook National University, Kyungpook National University Hospital, Daegu, South Korea.

This error has now been corrected in the HTML and PDF versions of the Article.

